# circHIPK3 Exacerbates Folic Acid-Induced Renal Tubulointerstitial Fibrosis by Sponging miR-30a

**DOI:** 10.3389/fphys.2021.715567

**Published:** 2022-01-04

**Authors:** Yan Wu, Junjun Luan, Congcong Jiao, Shiwen Zhang, Cong Ma, Yixiao Zhang, Jingqi Fu, En Yin Lai, Jeffrey B. Kopp, Jingbo Pi, Hua Zhou

**Affiliations:** ^1^Department of Nephrology, Shengjing Hospital of China Medical University, Shenyang, China; ^2^Department of Critical Care Medicine, Fushun Central Hospital, Fushun, China; ^3^Department of Urology, Shengjing Hospital of China Medical University, Shenyang, China; ^4^Program of Environmental Toxicology, School of Public Health, China Medical University, Shenyang, China; ^5^Department of Physiology, School of Basic Medical Sciences, Zhejiang University School of Medicine, Hangzhou, China; ^6^Kidney Disease Section, NIDDK, NIH, Bethesda, MD, United States

**Keywords:** renal fibrosis, circHIPK3, miR-30a, TGF-β1, renal biopsy

## Abstract

Renal tubulointerstitial fibrosis is a common pathological feature of progressive chronic kidney disease (CKD), and current treatment has limited efficacy. The circular RNA circHIPK3 is reported to participate in the pathogenesis of various human diseases. However, the role of circHIPK3 in renal fibrosis has not been examined. In this study, we aimed to determine whether and how circHIPK3 might participate in the pathogenesis of renal fibrosis. Mice received a peritoneal injection of folic acid (250 mg/kg). Of note, 30 days later, renal fibrosis was present on periodic acid–Schiff (PAS) and Masson staining, and mRNA and protein of profibrotic genes encoding fibronectin (FN) and collagen 1 (COL1) were increased. Renal circHIPK3 was upregulated, while miR-30a was downregulated, assessed by quantitative PCR (qPCR) and fluorescence *in situ* hybridization (FISH). The expression of transforming growth factor beta-1 (TGF-β1) was increased by qPCR analysis, immunoblotting, and immunofluorescence. Renal circHIPK3 negatively correlated with miR-30a, and kidney miR-30a negatively correlated with TGF-β1. Target Scan and miRanda algorithms predicted three perfect binding sites between circHIPK3 and miR-30a. We found that circHIPK3, miR-30a, and TGF-β1 colocalized in the cytoplasm of human tubular epithelial cells (HK-2 cells) on FISH and immunofluorescence staining. We transfected circHIPK3 and a scrambled RNA into HK-2 cells; miR-30a was downregulated, and the profibrotic genes such as TGF-β1, FN, and COL1 were upregulated and assessed by qPCR, immunoblotting, and immunofluorescence staining. Third, the upregulation of circHIPK3, downregulation of miR-30a, and overproduction of profibrotic FN and COL1 were also observed in HK-2 cells exposed to TGF-β1. Finally, renal biopsies from patients with chronic tubulointerstitial nephritis manifested similar expression patterns of circHIPK3, miR-30a, and profibrotic proteins, such as TGF-β1, FN, and COL1 as observed in the experimental model. A feed-forward cycle was observed among circHIPK3, miR-30a, and TGF-β1. Our results suggest that circHIPK3 may contribute to progressive renal fibrosis by sponging miR-30a. circHIPK3 may be a novel therapeutic target for slowing CKD progression.

## Introduction

Renal tubulointerstitial fibrosis is a common pathologic feature of all chronic kidney diseases (CKDs), which affects 8–16% of the population worldwide and is a leading cause of death (Chen T. et al., 2019). At present, there are few effective therapies to prevent or retard the progression of tubulointerstitial fibrosis. Reducing the prevalence of CKD requires an in-depth understanding of the pathogenesis of tubulointerstitial fibrosis and the discovery of novel therapeutic agents targeting key mediators of tubulointerstitial fibrosis.

Circular RNA (circRNA) is a class of non-coding RNA molecules, produced with 3′ to 5′ back splicing, in which an upstream 3′ splicing site is joined with a downstream 5′ splicing site (Li et al., 2015). circRNAs participate in the pathogenesis of various human diseases by sponging microRNAs (miRNAs), consequently regulating gene expression at the transcriptional level (Dong et al., 2017; Li et al., 2017; Tan et al., 2017; Zhao et al., 2017). However, a possible interaction between circRNA and miRNA in renal tubulointerstitial fibrosis has not been explored.

Recently, circHIPK3 has been identified in several human and animal tissues (Zheng et al., 2016). circHIPK3 regulates cell growth, and migration by sponging multiple miRNAs, such as miR-124, miR-7, miR-30a, miR-193a, miR-338-3p, miR-107, miR-124, and miR-524 in cancers and non-kidney diseases (Shan et al., 2017; Chen et al., 2018; Liu et al., 2018; Zeng et al., 2018; Hong et al., 2020; Liu Z. et al., 2020; Shu et al., 2020; Yin and Cui, 2020). circHIPK3 promotes metastasis in gastric cancer *via* miR-653-5p/miR-338-3p (Jin et al., 2020) and by sponging miR-508-3p in clear cell renal cell carcinoma (Han et al., 2020).

The circHIPK3 is also involved in fibrosis in non-neoplastic tissues. circHIPK3 regulates cardiac fibroblast proliferation, migration, and phenotypic switching *via* the miR-152-3p/TGF-β2 axis under hypoxic conditions (Liu W. et al., 2020). The inhibition of circHIPK3 prevents angiotensin II-induced cardiac fibrosis by sponging miR-29b-3p (Ni et al., 2019). circHIPK3 regulates lung fibroblast-to-myofibroblast transition by competing with miR-338 (Zhang et al., 2019). Recently, circHIPK3 has been reported to exacerbate diabetic nephropathy and promote proliferation through sponging miR-185 (Liu et al., 2021). However, the role of circHIPK3 in the progression of renal tubulointerstitial fibrosis remains to be fully characterized.

This study aimed to determine whether circHIPK3 contributes to renal tubulointerstitial fibrosis and to identify the underlying mechanisms. In an experimental mouse model and in human tissues, we found that renal circHIPK3 was upregulated, while miR-30a was downregulated. Consequently, transforming growth factor beta-1 (TGF-β1) was overproduced. In a feed-forward loop, TGF-β1 induced an increase in circHIPK3.

## Materials and Methods

### Mouse Folic Acid-Induced Renal Tubulointerstitial Fibrosis Model

Male C57BL/6 mice (age = 16 weeks old, weight = 25–30 g, *n* = 12) were purchased from the Beijing Vital River Laboratory Animal Technology (Beijing, China). Mice were housed under standard conditions. The mice were randomly divided into two groups, namely, normal control (NC) group and FA group. FA was injected into the abdominal cavity of mice with a dosage of 250 mg/kg. Equal volumes of 0.3 mmol/L NaHCO_3_ were injected into the NC group. Of note, 30 days later, mice were weighed and euthanized with carbon dioxide. Kidneys were collected and placed at 80°C for further analysis. Animal studies were approved in advance by the Animal Care and Use Committee of China Medical University (15052111) and were performed following the NIH Animal Care and Use Guidelines.

### HK-2 Cell Culture and Transfection of circHIPK3 or Stimulation of Transforming Growth Factor Beta-1

Immortalized human renal proximal tubular epithelial cells (HK-2 cells) were purchased from ATCC (Manassas, VA, United States) and cultured in DMEM/F12 medium supplemented with 10% FBS at a culture density of 2 × 10^5^ cells in a six-well plate (Costar, Corning, NY, United States) at 37°C and 5% CO_2_. Cultured HK-2 cells were transfected with the circular sequence of circHIPK3 [pCDH-CMV-5′ circular Frame (1)-has_circHIPK3-3′ circular Frame(1)-EF1-copGFP-T2A-puro]/scrambled sequence [pCDH-CMV-5′circular Frame (1)-MCS-3′ circular Frame(1)-EF1-copGFP-T2A-puro] as negative control (SyngenTech, Beijing, China) using Lipofectamine 3000 (Invitrogen, CA, United States) for 48 h according to the instructions of the manufacturer. Cultured HK-2 cells were stimulated with hTGF-β (250 pg/ml) for 48 h. Cells were collected for RNA and protein extraction after the transfection of circHIPK3 or the stimulation of TGF-β1 for the analysis by quantitative PCR (qPCR) and Western blotting.

### Patients With Chronic Tubulointerstitial Nephritis

Patients with chronic tubulointerstitial nephritis on biopsy were from the Department of Nephrology at Shengjing Hospital of China Medical University between January 2019 and March 2020. Diagnoses were made by a nephropathologist following established criteria. Normal human kidney tissues were obtained at the time of renal cancer surgery. These tissues are located at least 5 cm away from the tumors as we previously described (Luan et al., 2018). All subjects provided written consent, and the research protocol was approved in advance by the Institutional Review Board of the China Medical University.

### Kidney Histology

Tissue sections (3 μm) were cut from paraffin-embedded human or mouse kidney blocks, deparaffinized, and rehydrated. Tissues were stained with periodic acid–Schiff (PAS) and Masson stains. The tubular injury was scored on PAS staining described previously, and the renal tubulointerstitial fibrosis area was presented as the percentage of the whole kidney cross-section on Masson staining using ImageJ software (NIH, Bethesda, MD, United States) on 15 randomly selected fields (200× magnification) as previously described (Liu et al., 2009; Luan et al., 2020).

### Quantitative PCR

Total RNA was extracted from frozen kidney tissue and HK-2 cells using TRIzol reagent (Life Technologies, Carlsbad, CA, United States), and the RNA concentration was determined using a NanoDrop 2000 spectrophotometer (ThermoFisher, Waltham, MA, United States). Total RNA (250 ng per sample) was subjected to reverse transcription using Prime Script RT Reagent Kit for circHIPK3, fibronectin (FN), collagen 1 (COL1), and TGF-β1 and to reverse transcription using TransScript miRNA First-Strand cDNA Synthesis SuperMix (TransGen Biotech, Beijing, China) for miR-30a and followed by PCR with SYBR Premix Ex Taq (Takara, Dalian, China). Real-time fluorescence was detected with QuantStudio 6 Flex quantitative PCR system (Applied Biosystems, Carlsbad, CA, United States). The relative expression level of each gene was expressed as 2^–ΔΔCt^ of each measurement. Glyceraldehyde-3-phosphate dehydrogenase (GAPDH), b-actin, or α-tubulin was used as an endogenous control for mRNAs and circHIPK3. Sno202 or U6 was used as an endogenous control for miR-30a. Primers were designed using Primer Express (Applied Biosystems, Carlsbad, CA, United States) and synthesized by Life Technologies (Shanghai, China) (Table 2).

**TABLE 1:** Clinical characteristics of human subjects.

No.	Age	Gender	eGFR (ml/min/1.73 m^2^)
1	50	F	139
2	58	M	127
3	70	M	94
4	67	F	46
5	46	M	66
6	37	M	50

*eGFR, estimated glomerular filtration rate based on the CKD-EPI formula. Cases 1–3, normal control kidney tissue from patients with a kidney tumor. Cases 4–6, renal biopsies from patients with chronic tubulointerstitial nephritis.*

**TABLE 2:** Sequences of primers used in real-time quantitative PCR (qPCR).

Gene	Forward (5′-3′)	Reverse (5′-3′)
FN (has)	CGGTGGCTGTCAGTCAAAG	AAACCTCGGCTTCCTCCATAA
FN (mus)	ATGTGGACCCCTCCTGATAGT	GCCCAGTGATTTCAGCAAAGG
COL1 (has)	GAGGGCCAAGACGAAGACATC	CAGATCACGTCATCGCACAAC
COL1 (mus)	GACATGTTCAGCTTTGTGGACCTC	GGGACCCTTAGGCCATTGTGTA
TGF-β1 (has)	CAATTCCTGGCGATACCTCAG	GCACAACTCCGGTGACATCAA
TGF-β1 (mus)	CCCGAAGCGGACTACTATGC	CATAGATGGCGTTGTTGCGG
circHIPK3 (has)	CCAGTGACAGTTGTGACAGCTACC	GCCAAACGTGCCTCGACCAAG
circHIPK3 (mus)	GGATCGGCCAGTCATGTATC	ACCGCTTGGCTATACTTTGA
GAPDH (has)	GGAGCGAGATCCCTCCAAAAT	GGCTGTTGTCATACTTCTCATGG
Actin (mus)	TTCCTTCTTGGGTATGGAAT	GAGCAATGATCTTGATCTTC
miR-30a (has/mus)	TGTAAACATCCTCGACTGGAAG	
U6 (has)	GCTTCGGCAGCACATATACTAAAAT	
Sno202 (mus)	GCTGTACTGACTTGATGAAAGTACT	

*FN, fibronectin; COL1, collagen 1; TGF-β1, transforming growth factor beta-1; GAPDH, glyceraldehyde-3-phosphate dehydrogenase.*

### Immunoblotting

Kidney samples and HK-2 cells were sonicated and resuspended in radioimmunoprecipitation assay buffer with protease inhibitors. Protein concentration was measured using a bicinchoninic acid assay kit. Equal amounts of protein were separated by SDS–PAGE and transferred into polyvinylidene difluoride (PVDF) membranes (Millipore Immobilon-P, Darmstadt, Germany). After blocking with 5% skimmed milk, membranes were incubated with primary antibodies against FN, COL1, and TGF-β1 at 4°C overnight. Following repeated washing, the membranes were incubated with horseradish peroxidase-conjugated secondary antibody for 1 h at room temperature. The antibody-antigen reactions were determined using a high-sig ECL Western blotting substrate and visualized by the Tanon 5500 imaging system. Protein loading variation was normalized by α-tubulin, β-actin, or GAPDH. For quantitative analysis, blot density was analyzed with NIH ImageJ software. The protein level is expressed as the ratio of blot density from individual protein to its housekeeping antibody. Antibody information is presented in Supplementary Table 1.

### Immunofluorescence Staining

Paraffin-embedded human and mouse kidney tissues were cut at 2-μm thickness, deparaffinized, and rehydrated. Antigens were retrieved, and non-specific binding was blocked. Kidney sections were incubated with antibodies directed against FN, COL1, and TGF-β1. Slides with HK-2 cells were incubated with these three antibodies at 4°C overnight, followed by incubation with Alexa-594/Alexa-488 donkey anti-rabbit/anti-mouse IgG (Supplementary Table 1) at room temperature for 1 h in the dark. After three washes with phosphate-buffered saline (PBS), slides were mounted with diamidino-phenyl indole (DAPI) for 10 min. Images were captured by immunofluorescence microscopy (Nikon, Tokyo, Japan). Specific staining for each target protein was quantified as integrated optical density expressed per unit area by Image-Pro Plus 6.0 (Media Cybernetics, MD, United States), as described previously (Luan et al., 2020; Qi et al., 2020).

### Fluorescence *in situ* Hybridization

Fluorescence *in situ* hybridization (FISH) was performed on kidney tissue and HK-2 cells following the protocol of the manufacturer. Paraffin-embedded human kidney tissue sections were cut at 4-μm thickness. Sections were deparaffinized, rehydrated, and digested with trypsin at 37°C for 30 min. Slides with kidney sections or cultured HK-2 cells were hybridized with a digoxigenin-horseradish peroxidase (DIG-HRP)-labeled oligonucleotide probe complementary to circHIPK3 or miR-30a at 37°C overnight, followed by incubation with anti-DIG-HRP (Servicebio, Wuhan, China) for 50 min, fluorescein isothiocyanate-tyramide signal amplification for 5 min, and DAPI to stain DNA for 5 min. Images were captured by immunofluorescence microscopy (Nikon, Tokyo, Japan) as described previously (Luan et al., 2020).

### Statistical Analyses

GraphPad Prism 8.0 software (GraphPad, San Diego, CA, United States) was used to perform statistical analyses. The quantitative data were expressed as mean ± SD. Differences between the two groups were analyzed by the *t*-test. Correlations between two variables were analyzed by Pearson’s linear correlation analysis. *p*-Value < 0.05 was accepted as statistically significant.

## Results

### Folic Acid-Induced Renal Tubulointerstitial Fibrosis in Mice

**FIGURE 1:**
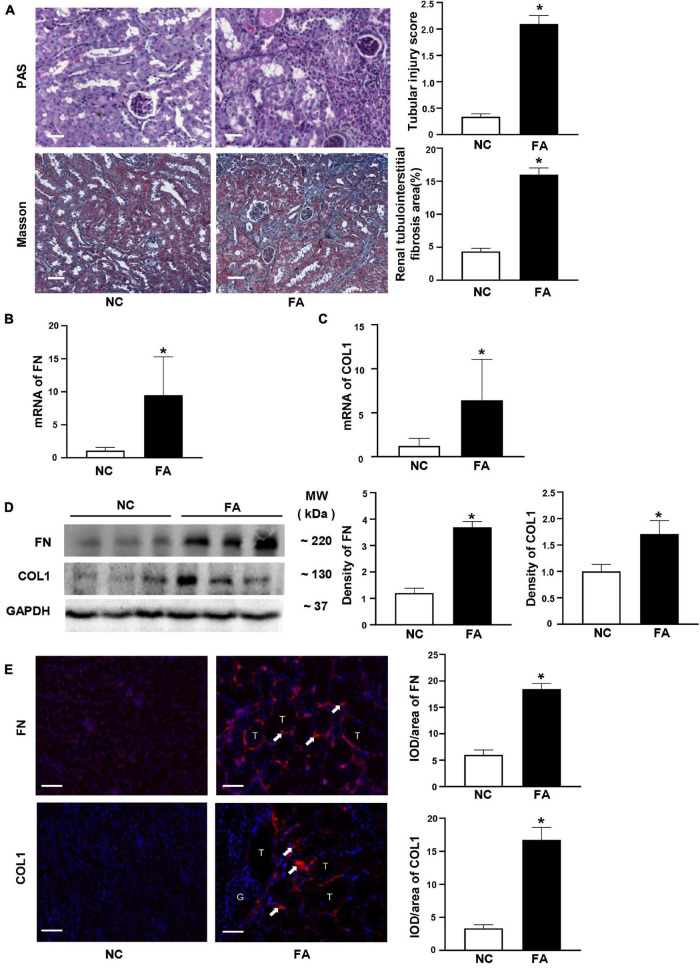
Typical renal fibrosis and the expression of fibronectin (FN)/collagen 1 (COL1) in folic acid (FA)-induced renal tubulointerstitial fibrosis in mice. **(A)** Periodic acid–Schiff (PAS) and Masson staining and semi-quantitative analysis. **(B,C)** mRNA levels of FN and COL1 on quantitative PCR (qPCR). **(D)** Proteins levels of FN and COL1 on immunoblotting and semi-quantitative analysis. **(E)** Expression of FN and COL1 (arrow) on immunofluorescent staining and semi-quantitative analysis. *n* = 6 in each group, *p < 0.05, FA vs. NC, magnification 200×, bar scale: 50 μm. FA, folic acid; RIF, renal tubulointerstitial fibrosis.

Mice with 250 mg/kg of FA administration developed renal tubulointerstitial fibrosis on day 30 after the injection. Collagen hyperplasia and inflammatory cell infiltration were shown in the tubulointerstitial area on PAS and Masson staining in FA mice. The tubular injury score and the area of renal fibrosis were elevated in the FA group compared to the NC group (Figure 1A). In the FA group, the mRNA levels of FN and COL1 were increased on qPCR (Figures 1B,C). The protein expression of FN and COL1 was increased by both Western blotting analysis (Figure 1D) and immunofluorescence staining (Figure 1E).

### Expression of Renal circHIPK3/miR-30a/Transforming Growth Factor Beta-1 in Folic Acid-Induced Renal Tubulointerstitial Fibrosis in Mice

**FIGURE 2:**
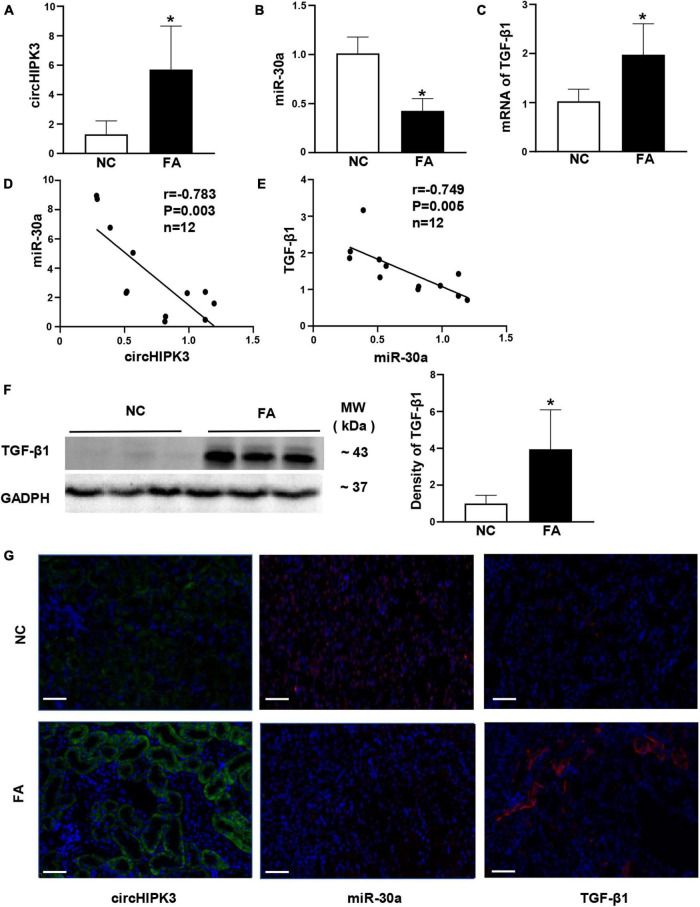
Expression of renal circHIPK3/miR-30a/transforming growth factor beta-1 (TGF-β1) in FA-induced renal tubulointerstitial fibrosis in mice. **(A–C)** circHIPK3, miR-30a, and TGF-β1 on qPCR. **(D,E)** The correlation between renal circHIPK3 and miR-30a as well as miR-30a and TGF-β1. **(F)** TGF-β1 on immunoblotting and semi-quantitative analysis. **(G)** circHIPK3 and miR-30a on fluorescence *in situ* hybridization (FISH) as well as TGF-β1 on immunofluorescence staining. *n* = 6 in each group, *p < 0.05, FA vs. NC, magnification 200×, bar scale: 50 μm. FA, folic acid; RIF, renal tubulointerstitial fibrosis.

Since circHIPK3 has been reported to participate in the fibrosis of the heart and lung in animal models, we investigated the expression of circHIPK3/miR-30a/TGF-β1 in mouse kidney tissue. We found that circHIPK3 was upregulated, while miR-30a expression was decreased, and TGF-β1 was increased (Figures 2A–C) in FA-induced renal tubulointerstitial fibrosis in mice compared to control mice. Importantly, circHIPK3 was negatively correlated with miR-30a, and miR-30a was also negatively correlated with TGF-β1 mRNA by the qPCR analysis (Figures 2D,E). TGF-β1 protein production was also increased in FA-induced renal tubulointerstitial fibrosis mice by immunoblotting (Figure 2F).

We used additional methods to evaluate the renal expression of circHIPK3/miR-30a/TGF-β1 in kidney tissue. FISH staining demonstrated that circHIPK3 and miR-30a are mainly located in tubules. Positive TGF-β1 staining located in tubules and tubulointerstitial area on immunostaining. The direction of renal circHIPK3/miR-30a/TGF-β1 was the same as qPCR or Western blotting in FA-treated mice compared to control mice (Figure 2G).

### Overexpression of circHIPK3 in HK-2 Cells

**FIGURE 3:**
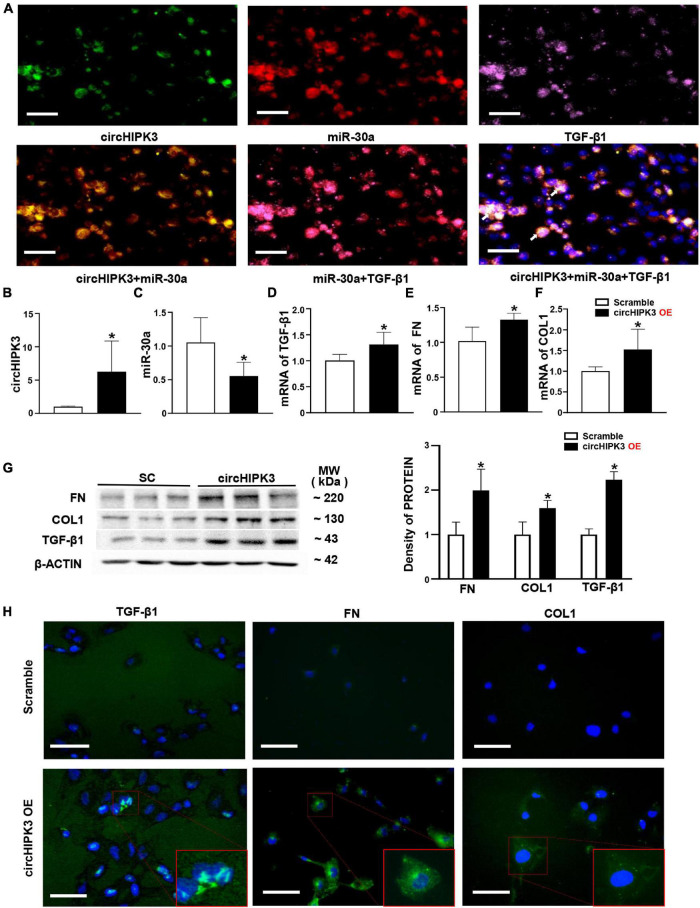
Overexpression of circHIPK3 in HK-2 cells. **(A)** The colocalization in the cytoplasm of circHIPK3, miR-30a, and TGF-β1 is shown. **(B)** The levels of circHIPK3 on qPCR after circHIPK3 overexpression. **(C–F)** The levels of miR-30a, TGF-β1, fibronectin (FN), and collagen 1 (COL1) using qPCR after overexpression of circHIPK3. **(G)** Protein levels of FN, COL1, and TGF-β1 on immunoblotting and semi-quantitative analysis after the overexpression of circHIPK3. **(H)** The expression of TGF-β1, FN, and COL1 assessed by immunofluorescence with circHIPK3 overexpression. Magnification 400×, bar scale: 100 μm. *n* = 6 in each group, *p < 0.05, circHIPK3 vs. scrambled RNA.

Based on the location of circHIPK3/miR-30a in FA-treated mice, we further utilized renal tubular cell line HK-2 cells to explore the roles of circHIPK3 in mice manifesting renal tubulointerstitial fibrosis. First, we found that circHIPK3, miR-30a, and TGF-β1 were mainly colocalized in the cytoplasm of normal HK-2 cells by triple staining, involving two FISH studies and one immunostaining (Figure 3A).

Then, we transfected the plasmid of circHIPK3 to upregulate circHIPK3 in HK-2 cells for 48 h. We found that the circHIPK3 level was nearly fivefold higher than that in the scrambled group (Figure 3B). Consequently, miR-30a was downregulated, and mRNA of TGF-β1 was upregulated on the qPCR analysis in HK-2 cells with the transfection of circHIPK3 compared to scrambled RNA sequence (Figures 3C,D). The expression changes of circHIPK3/miR-30a/TGF-β1 were similar to the changes of these genes in mouse kidney tissue. In addition, we examined two more profibrotic genes such as FN and COL1. We found that mRNAs of FN and COL1 were also upregulated in HK-2 cells with the overexpression of circHIPK3 (Figures 3E,F), similar to the TGF-β1 RNA expression change on qPCR. The protein levels of TGF-β1, FN, and COL1 were regulated similar to the RNA levels, which was confirmed by both Western blotting (Figure 3G) and immunofluorescence staining (Figure 3H) in HK-2 cells with the transfection of circHIPK3 compared to the scrambled RNA.

### Expression of circHIPK3/miR-30a/Profibrotic Genes in HK-2 Cells With Transforming Growth Factor Beta-1 Stimulation

**FIGURE 4:**
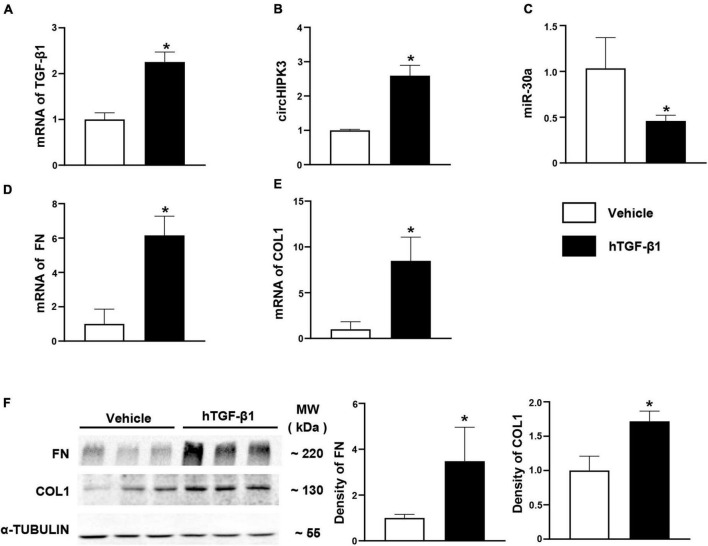
Expression of circHIPK3/miR-30a/TGF-β1 pathway in HK-2 cells with hTGF-β1 treated. **(A)** mRNA levels of TGF-β1 after hTGF-β1 stimulation in HK-2 cells. **(B–E)** The levels of circHIPK3, miR-30a, FN, and COL1 on qPCR after hTGF-β1 stimulation. **(F)** Protein levels of FN and COL1 on immunoblotting and semi-quantitative analysis after hTGF-β1 stimulation. *n* = 6 in each group, *p < 0.05, hTGF-β1 vs. vehicle.

The TGF-β1 is an important regulator of renal fibrosis and has been used to induce the overproduction of profibrotic proteins in human primary renal tubular epithelial cells *in vitro* studies from our group (Zhou H. et al., 2013) and others. In this study, we investigated the changes of circHIPK3/miR-30a/profibrotic genes such as FN and COL1 in HK-2 cells 48 h after exposure to human TGF-β1. In HK-2 cells, circHIPK3 level was increased 2.5-fold, and miR-30a expression was decreased by >50% with TGF-β1 stimulation compared to vehicle stimulant (Figures 4A–C). FN mRNA was also increased nearly sixfold, and COL1 mRNA was elevated by eightfold (Figures 4D,E). Consistent with mRNA expression changes of FN and COL1, protein expression was also increased in HK-2 cells following human TGF-β1 exposure compared to the vehicle, on the Western blot analysis (Figure 4F).

### Renal circHIPK3/miR-30a/Profibrotic Proteins in Human Subjects

**FIGURE 5:**
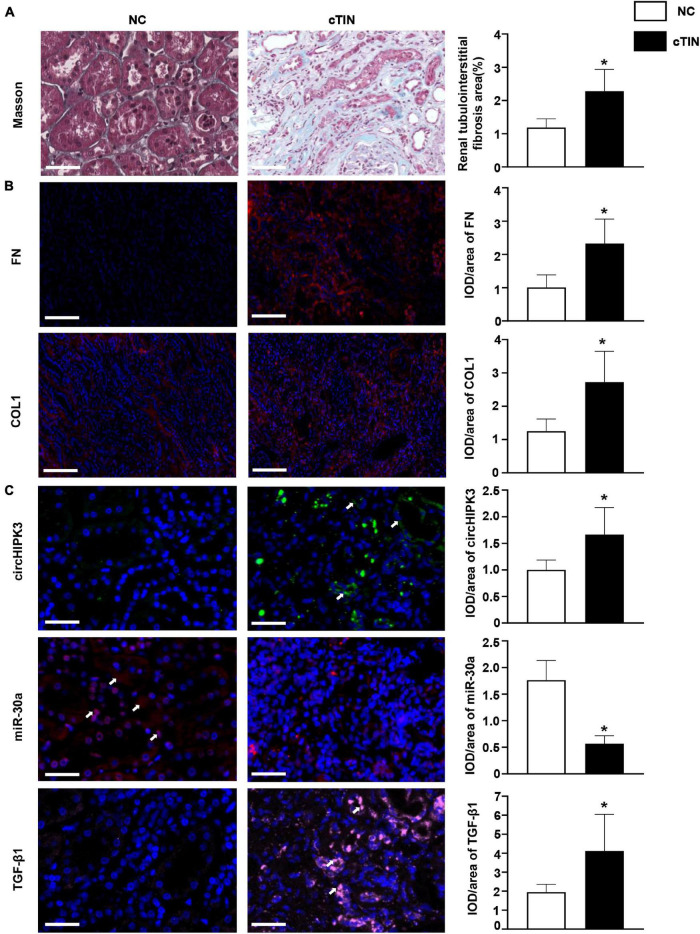
Renal expression of circHIPK3 in patients with chronic tubulointerstitial nephritis (cTIN). **(A)** Masson staining of kidney. **(B)** Expression of FN and COL1 shown by immunofluorescence staining. **(C)** Expression of circHIPK3, miR-30a, and TGF-β1 on FISH and immunofluorescence staining. Magnification 200×, bar scale: 50 μm. *n* = 3 in each group, *p < 0.05, cTIN vs. NC.

Finally, circHIPK3/miR-30a/profibrotic proteins were examined in kidney tissue from human subjects with chronic tubulointerstitial nephritis (cTIN) as relevant renal tubulointerstitial fibrosis tissue and normal tissue adjacent to kidney tumor as NC. The clinical characteristics of human subjects enrolled in the study are displayed in Table 1. Typical tubulointerstitial fibrosis was observed on Masson staining in renal biopsies from cTIN patients compared to normal human kidney tissues (Figure 5A). Fibrotic proteins FN and COL1 were increased in cTIN patients compared to NC on immunofluorescence staining (Figure 5B). The expression of circHIPK3 was upregulated, and miR-30a was downregulated, and excess TGF-β1 protein was present in renal biopsies from cTIN patients compared to NC kidney tissue on FISH or immunofluorescence staining (Figure 5C). It was consistent with the expression of circHIPK3/miR-30a/TGF-β1 in FA-induced renal tubulointerstitial fibrosis mice and HK-2 cells with circHIPK3 transfection.

## Discussion

The major findings of this study are as follows. First, renal circHIPK3 was upregulated, miR-30a was downregulated, and there was increased RNA expression of profibrotic genes, such as those encoding TGF-β1, FN, and COL1, in mice with FA-induced renal tubulointerstitial fibrosis and in renal biopsies from patients with cTIN. Second, the transfection of circHIPK3 to HK-2 resulted in the downregulation of miR-30a and the upregulation of TGF-β1, FN, and COL1. Third, circHIPK3, miR-30a, and TGF-β1 showed colocalization in normal HK-2 cells. Fourth, a feed-forward cycle was found among circHIPK3, miR-30a, and TGF-β1.

It has been established that circRNAs contribute to the initiation and progression of CKD. We have reported that circHLA-C plays an important role in lupus nephritis patients by regulating miR-150 (Luan et al., 2018). Exosomal circ_DLGAP4 promotes diabetic kidney disease progression by sponging miR-143 (Bai et al., 2020). circRNA_010383 acts as a sponge for miR-135a and contributes to renal fibrosis in experimental diabetic nephropathy (Peng et al., 2020).

The circHIPK3, derived from exon2 of the HIPK3 gene, is highly conserved in the genomes of mice, rats, and humans. circHIPK3 plays an important role in heart and lung fibrosis. circHIPK3 regulates cardiac fibrosis through sponging miR-152-3p and miR-29b-3p, respectively (Ni et al., 2019; Liu W. et al., 2020). circHIPK3 also regulates lung fibroblast-to-myofibroblast transition by miR-338 (Zhang et al., 2019). Most recently, circHIPK3 has been reported to be upregulated in early-phase diabetic nephropathy and to promote mesangial proliferation through sponging miR-185 (Liu et al., 2021). However, the role of circHIPK3 in renal fibrosis has not been reported.

In this study, we found that circHIPK3 was overexpressed in the late phase of experimental renal tubulointerstitial fibrosis induced by FA, which initiates renal tubular injury. Meanwhile, miR-30a was downregulated, and the profibrotic genes such as those encoding TGF-β1, FN, and COL1 were upregulated (Figure 1 and Supplementary Figure 1). These data demonstrate that circHIPK3 contributes to the development and progression of FA-induced renal fibrosis.

What mechanisms are involved in circHIPK3-mediated FA-induced renal fibrosis? Several studies have shown that circHIPK3 exerts various biological functions by acting as a sponge with multiple RNAs (Zheng et al., 2016). As a competitive endogenous RNA, circHIPK3 regulates cell growth and migration by sponging miR-30a in retinal vascular (Shan et al., 2017). circHIPK3 and miR-30a have three perfect match seeds (Chen B. et al., 2019), and miR-30a binds the 3′UTR of TGF-β1 (Bogusławska et al., 2018).

We asked whether circHIPK3 participates in the pathogenesis of FA-induced renal fibrosis by sponging miR-30a. We performed the triple localization of circHIPK3, miR-30a, and TGF-β1 and showed the coexpression of three molecules in the cytoplasm of normal HK-2 cells; this suggested that three molecules interact. This hypothesis is supported by the reports of these three genes being coexpressed in various cell types (Bogusławska et al., 2018; Chen B. et al., 2019).

To experimentally verify whether the circHIPK3/miR-30a/TGF-β1 axis contributes to renal fibrosis, we transfected circHIPK3 into HK-2 cells. We found that the overexpression of circHIPK3 downregulated miR-30a and stimulated overproduced TGF-β1, FN, and COL1 on either mRNA or protein levels (Figure 3). The expression of the circHIPK3/miR-30a/TGF-β1 pathway was also observed in renal biopsies from patients with cTIN, i.e., a renal tubular injury initiated clinical renal fibrosis disease (Figure 5). These results provided that more evidence of circHIPK3/miR-30a/TGF-β1 initiates tubular injury and thereby contributes to FA-induced renal tubulointerstitial fibrosis.

The TGF-β1 is a major driver of tissue fibrosis, and its protein expression is regulated by miR-30a in various cell types (Bogusławska et al., 2018). In the rat peritoneal fibrosis model, miR-30a expression is negatively correlated with TGF-β1 expression. The overexpression of miR-30a blocks TGF-β1-induced peritoneal fibrosis *via* inhibiting EMT and collagen production (Zhou Q. et al., 2013). In carbon tetrachloride-induced rat liver fibrosis, miR-30a serves as a crucial suppressor of TGF-β1 signaling in hepatic stellate cells activation (Zheng et al., 2018). Similarly, we found that the downregulation of miR-30a by circHIPK3 sponging resulted in the overexpression of TGF-β1 and profibrotic proteins, such as FN and COL1. Taken together, these results from diverse models provide compelling evidence that the circHIPK3/miR-30a/TGF-β1 axis contributes to the pathogenesis of renal fibrosis.

**FIGURE 6:**
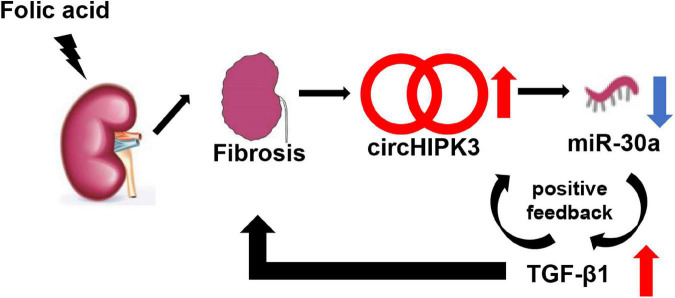
Schematic mechanism model of the role of circHIPK3. In FA-induced renal tubulointerstitial fibrosis, circHIPK3 is increased, miR-30a is decreased, and TGF-β1 is upregulated. A TGF-β1 feed-forward loop exacerbates the development of renal fibrosis.

*In vitro*, we studied TGF-β1-stimulated HK-2 cells and found that TGF-β1 stimulation increases circHIPK3, decreases miR-30a, and promotes the production of profibrotic proteins, such as FN and COL1. This suggested that a positive feedback loop may exist among circHIPK3, miR-30a, and TGF-β1 in renal fibrosis (Figure 6). Similar positive feedback has also been noted in miR-150 and TGF-β1 in human primary proximal tubular cells in our previous study (Zhou H. et al., 2013). Renal fibrosis is the characteristic of progressive CKD, and many pathways may contribute among them being circHIPK3/miR-30a/TGF-β1. Future studies might extend this study by using circHIPK3 knockout mice or using circHIPK3 RNAi delivery.

## Conclusion

The data presented in this study suggest that circHIPK3 contributes to the pathogenesis of renal fibrosis by sponging miR-30a and thereby increasing the production of profibrotic TGF-β1 protein. circHIPK3 may be a novel therapeutic target for renal fibrosis.

## Data Availability Statement

The original contributions presented in the study are included in the article/Supplementary Material, further inquiries can be directed to the corresponding authors.

## Ethics Statement

The studies involving human participants were reviewed and approved by the Institutional Review Board of Shengjing Hospital of China Medical University. The patients/participants provided their written informed consent to participate in this study. The animal study was reviewed and approved by Animal Care and Use Committee of China Medical University.

## Author Contributions

YW conceived and supervised the study. YW and JL designed the experiments and wrote the manuscript. YW, JL, CJ, SZ, and CM performed the experiments. JF, JP, and HZ cosupervised the work process. YW and YZ provided renal tissues and the clinical data of patients. YW, JL, and CJ analyzed the data. EL, JK, and HZ revised the manuscript. All authors contributed to the article and approved the submitted version.

## Conflict of Interest

The authors declare that the research was conducted in the absence of any commercial or financial relationships that could be construed as a potential conflict of interest.

## Publisher’s Note

All claims expressed in this article are solely those of the authors and do not necessarily represent those of their affiliated organizations, or those of the publisher, the editors and the reviewers. Any product that may be evaluated in this article, or claim that may be made by its manufacturer, is not guaranteed or endorsed by the publisher.
