# Supplementary material for: circHIPK3 Exacerbates Folic Acid-Induced Renal Tubulointerstitial Fibrosis by Sponging miR-30a
Source: Front Physiol. 2022 Jan 4;12:715567. doi: 10.3389/fphys.2021.715567 (PMC8763699; doi:10.3389/fphys.2021.715567)
Supplement: Supplementary file 1 [file Presentation_1.PPT]

## Slide 1
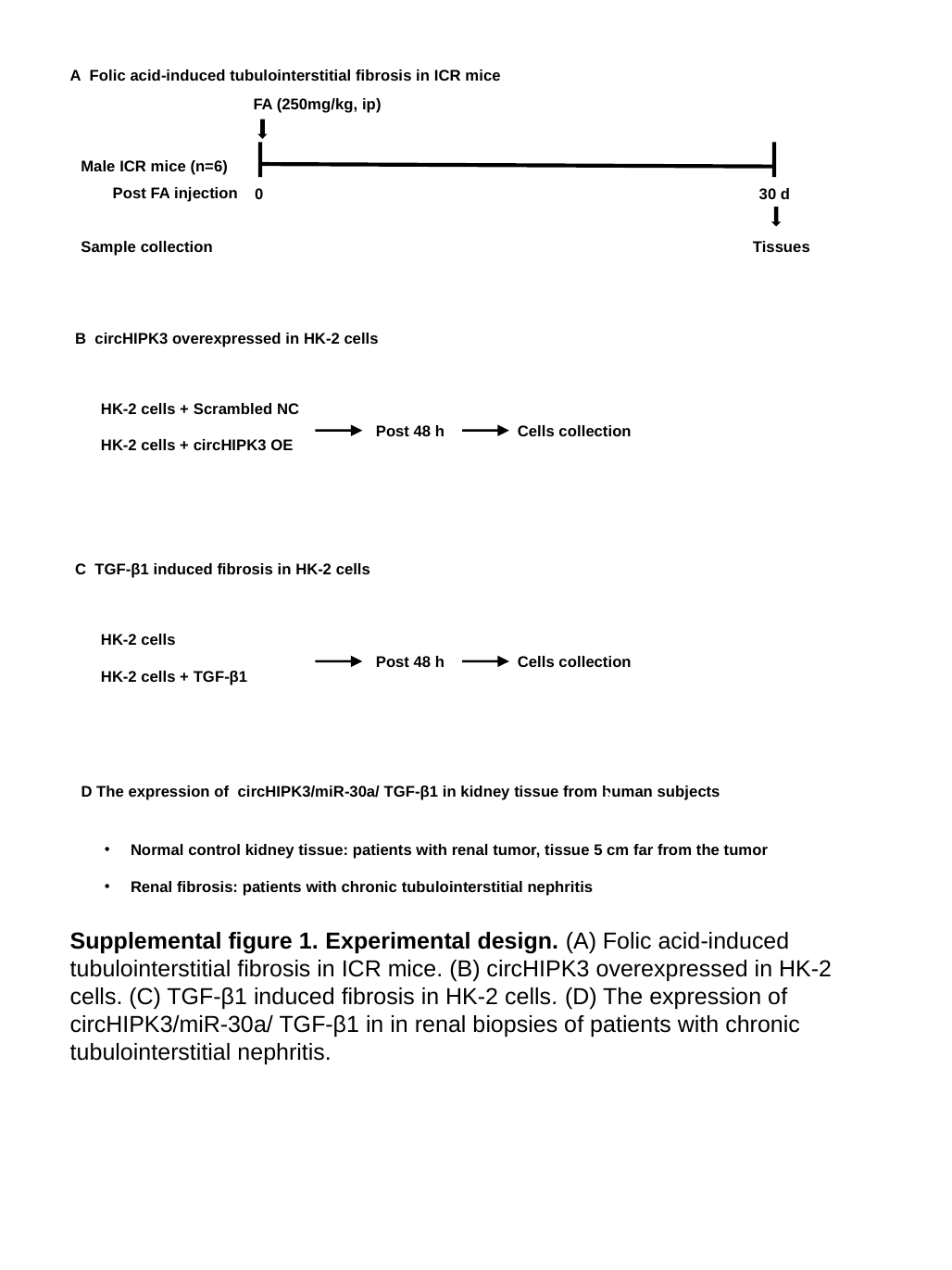

A Folic acid-induced tubulointerstitial fibrosis in ICR mice
FA (250mg/kg, ip)
Male ICR mice (n=6)
Post FA injection
0 30 d
Sample collection Tissues
B circHIPK3 overexpressed in HK-2 cells
HK-2 cells + Scrambled NC
Post 48 h
Cells collection
HK-2 cells + circHIPK3 OE
C TGF-β1 induced fibrosis in HK-2 cells
HK-2 cells
Post 48 h
Cells collection
HK-2 cells + TGF-β1
D The expression of circHIPK3/miR-30a/ TGF-β1 in kidney tissue from human subjects
Normal control kidney tissue: patients with renal tumor, tissue 5 cm far from the tumor
Renal fibrosis: patients with chronic tubulointerstitial nephritis
Supplemental figure 1. Experimental design. (A) Folic acid-induced tubulointerstitial fibrosis in ICR mice. (B) circHIPK3 overexpressed in HK-2 cells. (C) TGF-β1 induced fibrosis in HK-2 cells. (D) The expression of circHIPK3/miR-30a/ TGF-β1 in in renal biopsies of patients with chronic tubulointerstitial nephritis.

## Slide 2
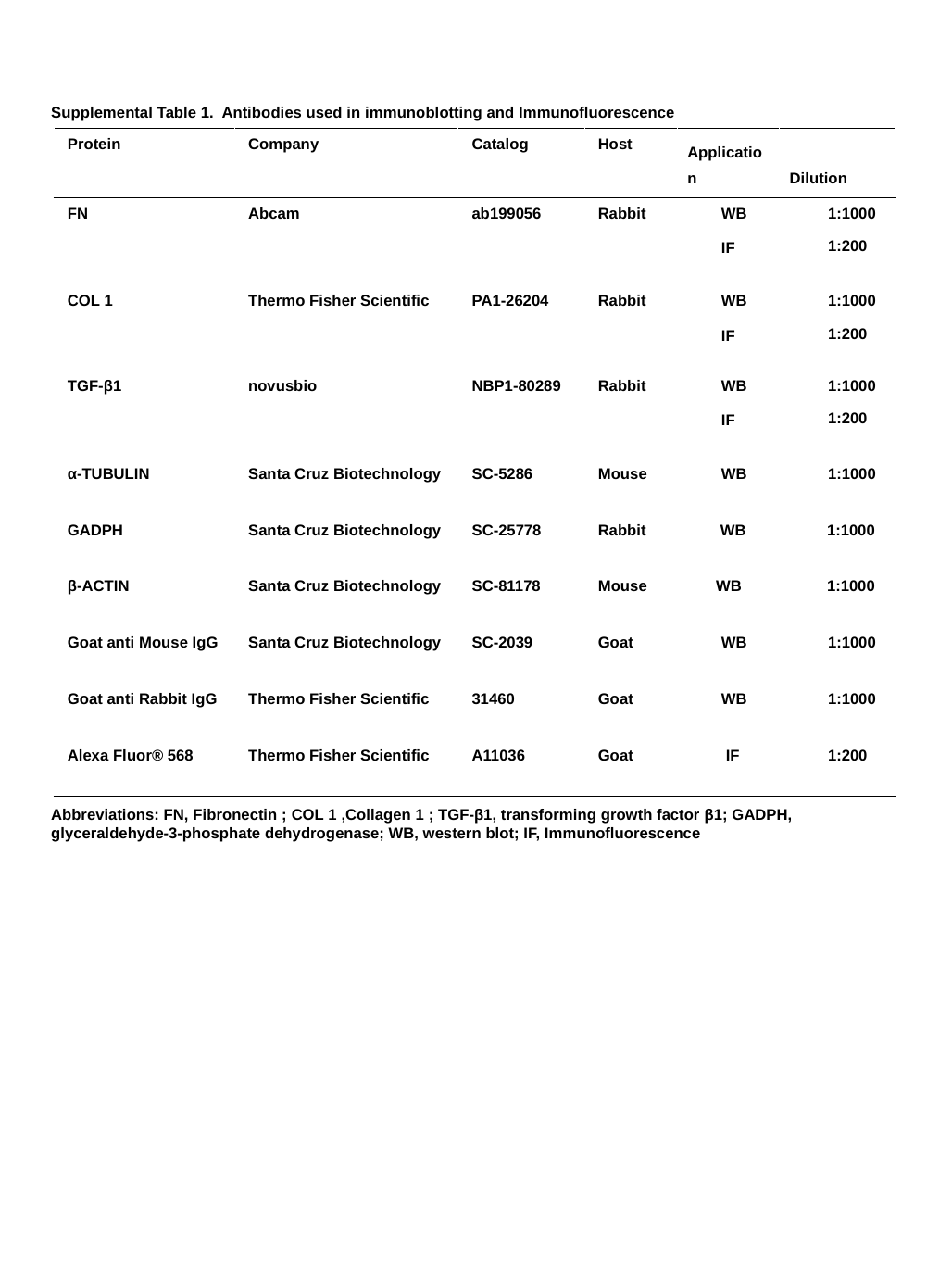

Supplemental Table 1. Antibodies used in immunoblotting and Immunofluorescence
| Protein | Company | Catalog | Host | Application | Dilution |
| --- | --- | --- | --- | --- | --- |
| FN | Abcam | ab199056 | Rabbit | WB IF | 1:1000 1:200 |
| COL 1 | Thermo Fisher Scientific | PA1-26204 | Rabbit | WB IF | 1:1000 1:200 |
| TGF-β1 | novusbio | NBP1-80289 | Rabbit | WB IF | 1:1000 1:200 |
| α-TUBULIN | Santa Cruz Biotechnology | SC-5286 | Mouse | WB | 1:1000 |
| GADPH | Santa Cruz Biotechnology | SC-25778 | Rabbit | WB | 1:1000 |
| β-ACTIN | Santa Cruz Biotechnology | SC-81178 | Mouse | WB | 1:1000 |
| Goat anti Mouse IgG | Santa Cruz Biotechnology | SC-2039 | Goat | WB | 1:1000 |
| Goat anti Rabbit IgG | Thermo Fisher Scientific | 31460 | Goat | WB | 1:1000 |
| Alexa Fluor® 568 | Thermo Fisher Scientific | A11036 | Goat | IF | 1:200 |
Abbreviations: FN, Fibronectin ; COL 1 ,Collagen 1 ; TGF-β1, transforming growth factor β1; GADPH, glyceraldehyde-3-phosphate dehydrogenase; WB, western blot; IF, Immunofluorescence
